# Molecular and macromolecular alterations of recombinant adenoviral vectors do not resolve changes in hepatic drug metabolism during infection

**DOI:** 10.1186/1743-422X-5-111

**Published:** 2008-09-30

**Authors:** Shellie M Callahan, Piyanuch Wonganan, Maria A Croyle

**Affiliations:** 1 College of Pharmacy, Division of Pharmaceutics, The University of Texas at Austin, Austin, TX, USA; 2 Institute of Cellular and Molecular Biology, The University of Texas at Austin, Austin, TX, USA

## Abstract

In this report we test the hypothesis that long-term virus-induced alterations in CYP occur from changes initiated by the virus that may not be related to the immune response. Enzyme activity, protein expression and mRNA of CYP3A2, a correlate of human CYP3A4, and CYP2C11, responsive to inflammatory mediators, were assessed 0.25, 1, 4, and 14 days after administration of several different recombinant adenoviruses at a dose of 5.7 × 10^12 ^virus particles (vp)/kg to male Sprague Dawley rats. Wild type adenovirus, containing all viral genes, suppressed CYP3A2 and 2C11 activity by 37% and 39%, respectively within six hours. Levels fell to 67% (CYP3A2) and 79% (CYP2C11) of control by 14 days (*p *≤ 0.01). Helper-dependent adenovirus, with all viral genes removed, suppressed CYP3A2 (43%) and CYP2C11 (55%) within six hours. CYP3A2 remained significantly suppressed (47%, 14 days, *p *≤ 0.01) while CYP2C11 returned to baseline at this time. CYP3A2 and 2C11 were reduced by 45 and 42% respectively 6 hours after treatment with PEGylated adenovirus, which has a low immunological profile (*p *≤ 0.05). CYP3A2 remained suppressed (34%, *p *≤ 0.05) for 14 days while CYP2C11 recovered. Inactivated virus suppressed CYP3A2 activity by 25–50% for 14 days (*p *≤ 0.05). CYP2C11 was affected similar manner but recovered by day 14. Microarray and *in vitro *studies suggest that changes in cellular signaling pathways initiated early in virus infection contribute to changes in CYP.

## Introduction

Hepatic cytochrome P450 (CYP) enzymes play a central role in the metabolism and clearance of many naturally occurring biological substances, drugs and environmental toxins [1,2]. In turn, their diversity, expression and function may also be modified by these substrates [3,4]. Numerous clinical reports have also described altered pharmacokinetic and toxicity profiles of drugs during infection or inflammation [5,6]. In these instances, the activity and expression of CYP is downregulated, leading to ineffective treatment regimens, unexpected adverse reactions and in, some cases, drug-drug interactions [7,8]. Similar effects have been reported with respect to the expression and function of CYP isforms 3A2 and 2C11 after a single dose of recombinant adenovirus serotype 5 for a period of 14 days in the male Sprague Dawley rat [9,10]. The expression and function of these isoforms, selected for their predominance in drug metabolism (CYP3A2) and their responsiveness to inflammatory stimuli (CYP2C11), are largely influenced by the dose (5.7 × 10^6 ^– 5.7 × 10^12 ^viral particles/kilogram (vp/kg)) and the nature of the transgene cassette. Although much is known about the regulatory processes associated with CYP3A2 and 2C11 expression, the exact mechanism by which virus infection alters these metabolic enzymes is currently unknown.

Recombinant adenoviruses were chosen as model pathogens to further define processes by which viral infection alters expression of CYP3A2 and 2C11 for several reasons. Although wild type adenovirus infections are common in the general population and often cause self-limited respiratory infections, they also induce significant illness and high mortality in specialized patient populations such as those receiving allogenic stem cell and solid organ transplants and those with acute respiratory illnesses [11-14]. Within the last decade, extensive use of adenovirus serotype 5 as a vector for gene therapy and vaccine development has increased understanding of the biology and the genetic features of the virus. This information has fostered the production of a series of recombinant viruses with minimal viral elements to reduce the host immune response and extend the length of gene expression achieved by this otherwise highly efficient vector. In this report, a panel of recombinant adenoviruses were studied in a Sprague Dawley rat model to test the hypothesis that changes in hepatic CYP expression and function after a single systemic dose of virus may not be solely due to the immune response against viral gene products and capsid proteins. Wild type adenovirus serotype 5, capable of causing mild illness in the general public and more severe complications in the immunosuppressed and those with asthma and COPD, was used as a positive control. It contained all virus expression elements. A first generation adenoviral vector, expressing the *E. coli *beta-galactosidase transgene (AdlacZ) was included as an important control for direct comparison of results previously reported to those obtained from animals treated with the other modified vectors [9,10]. The early region 1 (E1, involved in virus replication) and early region 3 (E3, involved in evasion of the host immune response) parts of the virus genome were removed in this vector to accommodate the beta-galactosidase transgene cassette. A PEGylated version of this virus, which has a significantly lower immunological profile [15,16], and an inactive control, AdlacZ inactivated by exposure to riboflavin and UV light, were included to study the effect of the immune response against virus capsid proteins and virus receptor interactions on expression and function of CYP. A helper-dependent adenoviral vector (HDAd), devoid of all viral genes and containing the *E. coli *beta-galactosidase transgene, was also included to fully study the effect of viral gene expression on CYP. CYP protein expression, activity, and mRNA were evaluated at 0.25, 1, 4 and 14 days, a time course highlighting key points during adenoviral gene expression and the host immune response [17]. Serum alanine amintotransferase levels and histological evaluation of liver tissue were used to evaluate the toxicity associated with administration of each vector. Expression patterns of the pregnane × receptor (PXR) and the retinoid × receptor alpha (RXR∝), involved in transcriptional regulation of CYP [18,19], and those associated with several signal transduction pathways in the liver are also discussed.

## Results

### Effect of administration of recombinant adenoviruses on hepatic CYP3A2 expression and function

**Figure 1:**
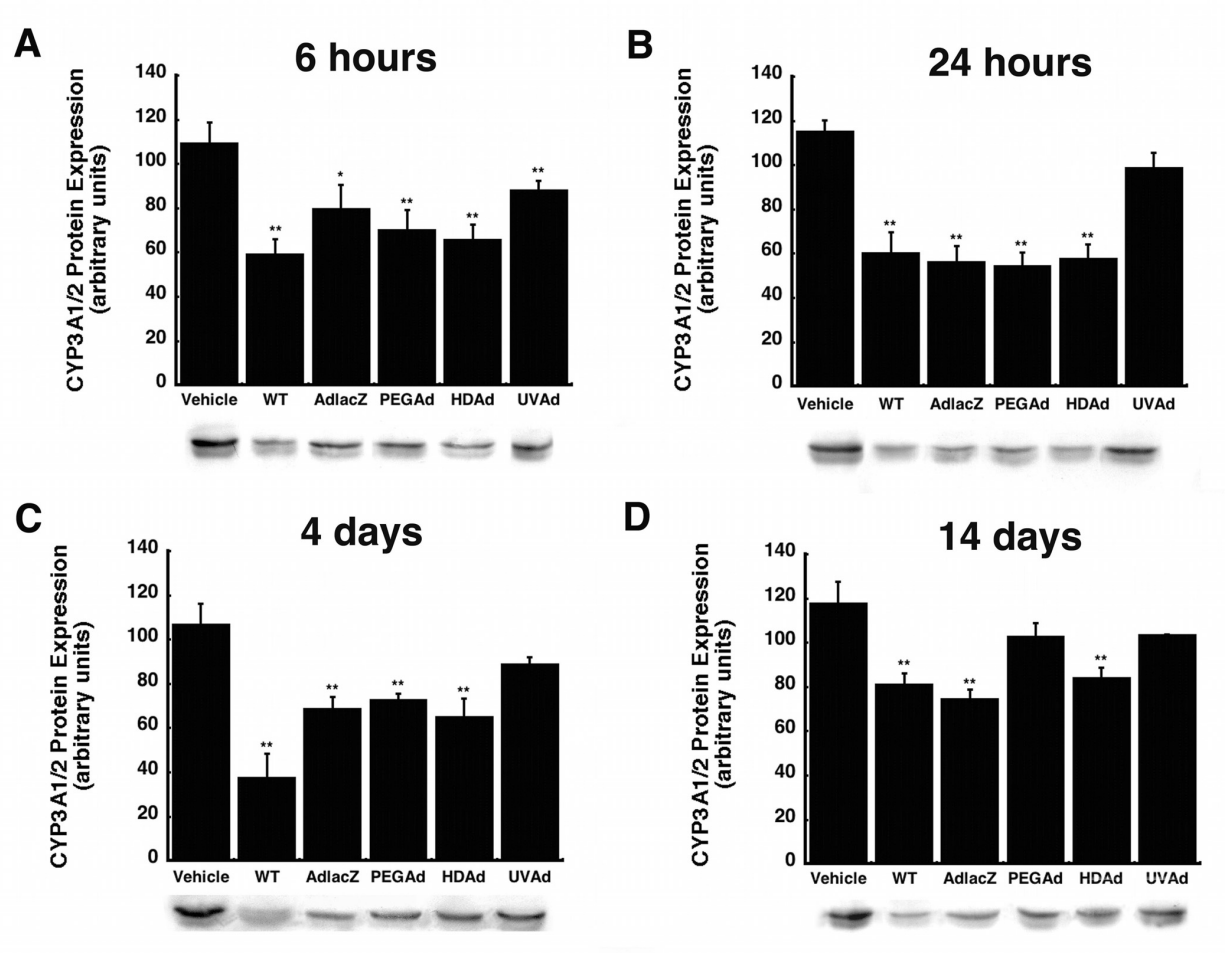
**Administration of a Single Dose of Active Adenovirus Significantly Suppresses Hepatic CYP3A1/2 Protein Expression for 14 Days without Recovery**. Immunoblot analysis and representative immunoblots of hepatic CYP3A1/2 protein expression 0.25 (A), 1 (B), 4 (C), and 14 (D) days after a single intravenous dose of wild type adenovirus serotype 5 (WT), a first generation recombinant adenovirus serotype 5 expressing *E. coli *beta-galactosidase (AdlacZ), PEGylated AdlacZ, (PEGAd), helper-dependent adenovirus serotype 5 expressing beta-galactosidase (HDAd), or inactivated AdlacZ (UVAd) in male Sprague-Dawley rats. Protein levels are reported as the relative density of positive bands with respect to that of a known protein standard in arbitrary units. Values are presented as the mean ± standard error of 4 animals/treatment/timepoint. Statistical significance was determined between individual treatment groups and vehicle controls by one-way analysis of variance with a Bonferroni/Dunn post-hoc analysis. *p ≤ 0.05, **p ≤ 0.01.

Hepatic CYP3A2 protein expression, catalytic activity, and mRNA levels were analyzed at 0.25, 1, 4, and 14 days after administration of either wild type virus or several different recombinant adenoviruses. Six hours after administration, each virus significantly suppressed CYP3A1/2 protein (Figure 1A). The most pronounced effect was observed in samples obtained from animals given wild type (WT) virus with protein levels 46% of those seen in saline treated animals (Vehicle, *p *≤ 0.01). Inactivated virus (UVAd) affected CYP3A1/2 protein the least (19% of control, *p *≤ 0.01). Twenty-four hours after treatment, protein levels of these animals returned to baseline and remained so for the duration of the study (Figure 1B). At this time, the other vectors reduced CYP3A1/2 by approximately 50% (*p *≤ 0.01). Four days after treatment, CYP3A1/2 was still markedly suppressed in animals given WT virus, 65% of control, but levels began to recover in animals given the other viruses. Protein expression levels were 36%, 32%, and 39% of control for animals given AdlacZ, PEGAd, and HDAd, respectively at this timepoint (Figure 1C, *p *≤ 0.01). Fourteen days after administration CYP3A1/2 protein remained significantly suppressed without evidence of recovery in animals treated with WT (31% of control) AdlacZ (37%) and HDAd (29%) (Figure 1D, *p *≤ 0.01).

**Figure 2:**
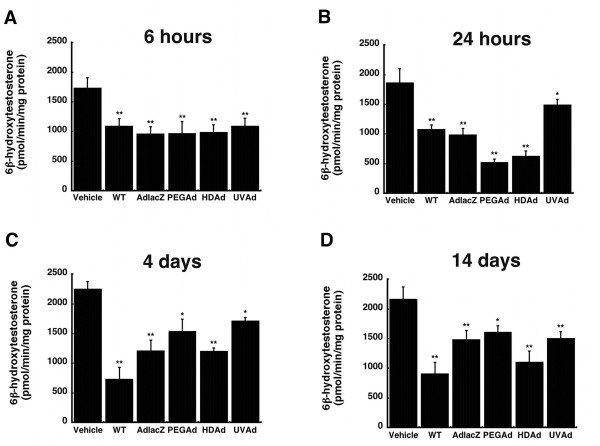
**Administration of a Single Dose of Active and Inactive Adenovirus Significantly Reduces Hepatic CYP3A2 Activity in the Male Sprague-Dawley Rat for 14 Days without Return to Baseline Levels**. *In vitro *catalytic activity of CYP3A2 microsomal proteins were measured by the production of the enzyme-specific testosterone metabolite, 6β-hydroxytestosterone. Rats were treated with: phosphate buffered saline (Vehicle), wild type adenovirus serotype 5 (WT), a first generation recombinant adenovirus 5 expressing *E. coli *beta-galactosidase (AdlacZ), PEGylated AdlacZ, (PEGAd), helper-dependent adenovirus 5 expressing beta-galactosidase (HDAd), or inactivated AdlacZ (UVAd). Values are presented as the mean ± standard error of 4 animals/treatment/timepoint. Statistical significance was determined between individual treatment groups and saline controls by one-way analysis of variance with a Bonferroni/Dunn post-hoc analysis. *p ≤ 0.05, **p ≤ 0.01.

CYP3A2 activity was assessed by separation and quantification of the isoform-specific primary testosterone metabolite, 6β-hydroxytestosterone. Each virus significantly reduced CYP3A2 activity throughout the entire duration of the study (Figure 2). Six hours after administration, CYP3A2 activity was suppressed by approximately 41%, in each treatment group with respect to that of saline treated animals (Figure 2A, *p *≤ 0.01). Twenty-four hours after administration, the most significant suppression was seen in samples obtained from animals treated with PEGAd, 72% of control, and HDAd, 67% (*p *≤ 0.01). Both WT and AdlacZ treated animals experienced a notable reduction in metabolic activity, approximately 45%. CYP3A2 activity was reduced by 20% in animals given the UVAd vector at the same timepoint (Figure 2B, *p *≤ 0.01). In a manner similar to protein expression, the wild-type virus induced the most significant suppression of CYP3A2 activity four days after administration. Animals given this virus had activity levels that were 67% of that found in saline treated animals (Figure 2C, *p *≤ 0.01). CYP3A2 activity for both AdlacZ and UVAd treated animals were similar to that seen at 24 hours, 46% and 24% of control, respectively. At the same timepoint, activity levels for animals given either PEGAd or HDAd began to recover to baseline levels. Fourteen days after a single dose of virus, CYP3A2 activity continued to be reduced in each treatment group. Treatment with WT, AdlacZ, PEGAd, HDAd, and UVAd, resulted in activity that was 58%, 32%, 26%, 49%, and 31% of saline treated animals respectively (Figure 2D, *p *≤ 0.05).

**Figure 3:**
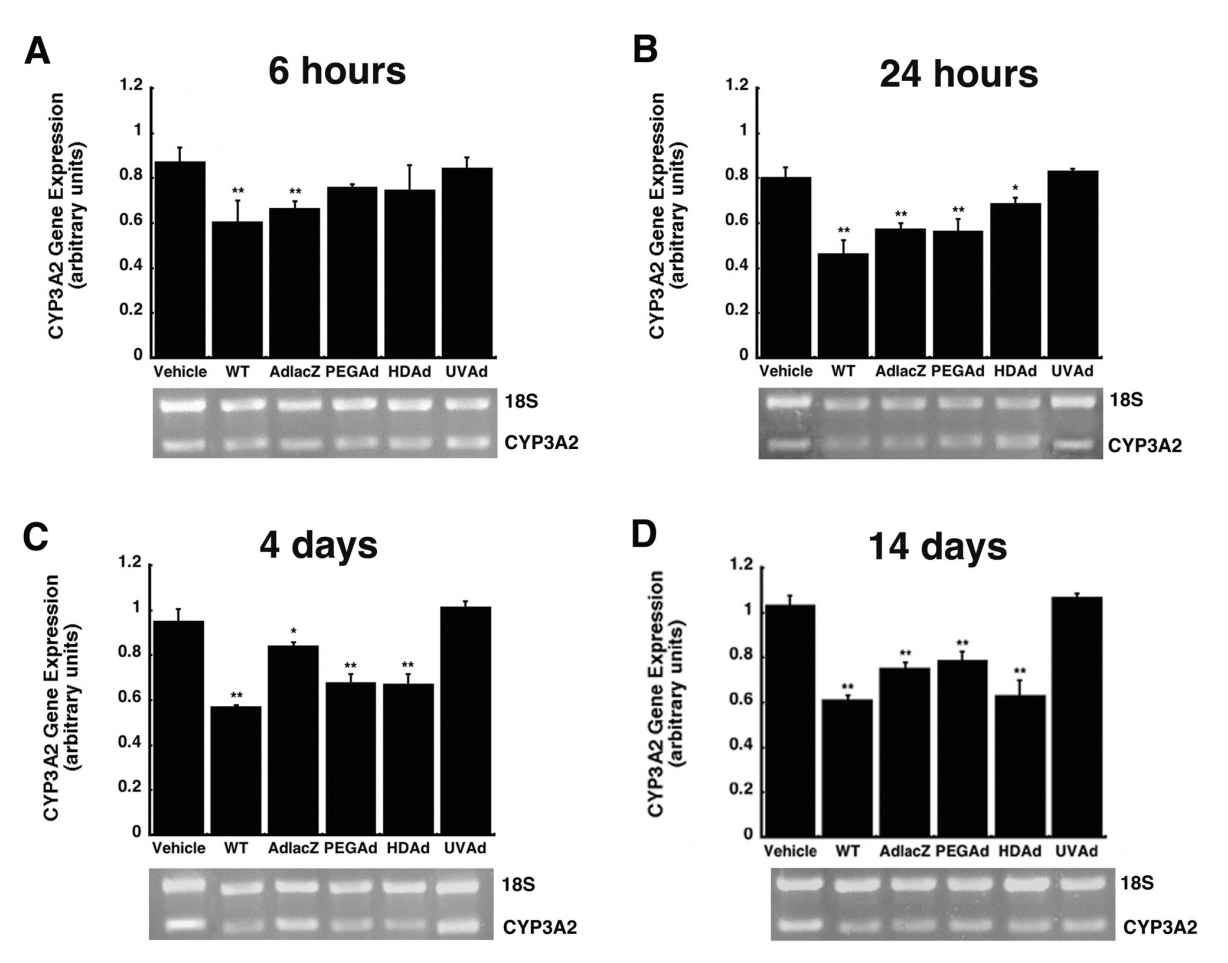
**A Single Dose of Active Virus Significantly Suppresses Hepatic CYP3A2 mRNA in Male Sprague Dawley Rats**. Mean relative intensities of CYP3A2 mRNA and representative agarose gels of PCR products 0.25 (A), 1 (B), 4 (C), and 14 (D) days after treatment with wild type adenovirus 5 (WT), first generation recombinant adenovirus expressing *E. coli *beta-galactosidase (AdlacZ), PEGylated AdlacZ, (PEGAd), helper-dependent virus expressing beta-galactosidase (HDAd), or inactivated AdlacZ (UVAd). mRNA levels are reported as band densities of gene-specific RT-PCR products with respect to the density of products from an internal control (18S rRNA) in arbitrary units. In all panels, animals dosed with phosphate buffered saline served as controls (Vehicle). Values are presented as the mean ± standard error of 4 animals/treatment/timepoint. Statistical significance was determined between individual treatment groups and vehicle controls by one-way analysis of variance with a Bonferroni/Dunn post-hoc analysis. *p ≤ 0.05, **p ≤ 0.01.

Administration of active viruses also significantly reduced hepatic CYP3A2 mRNA as early as six hours after administration. Treatment with WT and AdlacZ reduced mRNA levels by 31% and 24% respectively (Figure 3A, *p *≤ 0.01). CYP3A2 mRNA levels of animals treated with the wild-type virus were consistently suppressed throughout the duration of the study. They were reduced by 42%, 40% and 41% after 24 hours, 4, and 14 days (Figure 3B, C, and 3D, *p *≤ 0.01). Contrary to these findings, the UV inactivated virus did not alter CYP3A2 mRNA. Twenty-four hours after administration, mRNA levels in the AdlacZ and PEGAd treatment groups were approximately 30% below those of saline treated animals (Figure 3B, *p *≤ 0.01). At the four-day timepoint, mRNA levels remained suppressed in the PEGAd treated animals (29% of control). mRNA was reduced in a similar manner in animals treated with HDAd (30% of control, Figure 3C, *p *≤ 0.01). Fourteen days after administration, mRNA was still reduced in animals given AdlacZ, PEGAd, and HDAd (27, 27, and 39%, of control respectively, Figure 3D, *p *≤ 0.01).

### Effect of a single dose of virus on hepatic CYP2C11 expression and function

**Figure 4:**
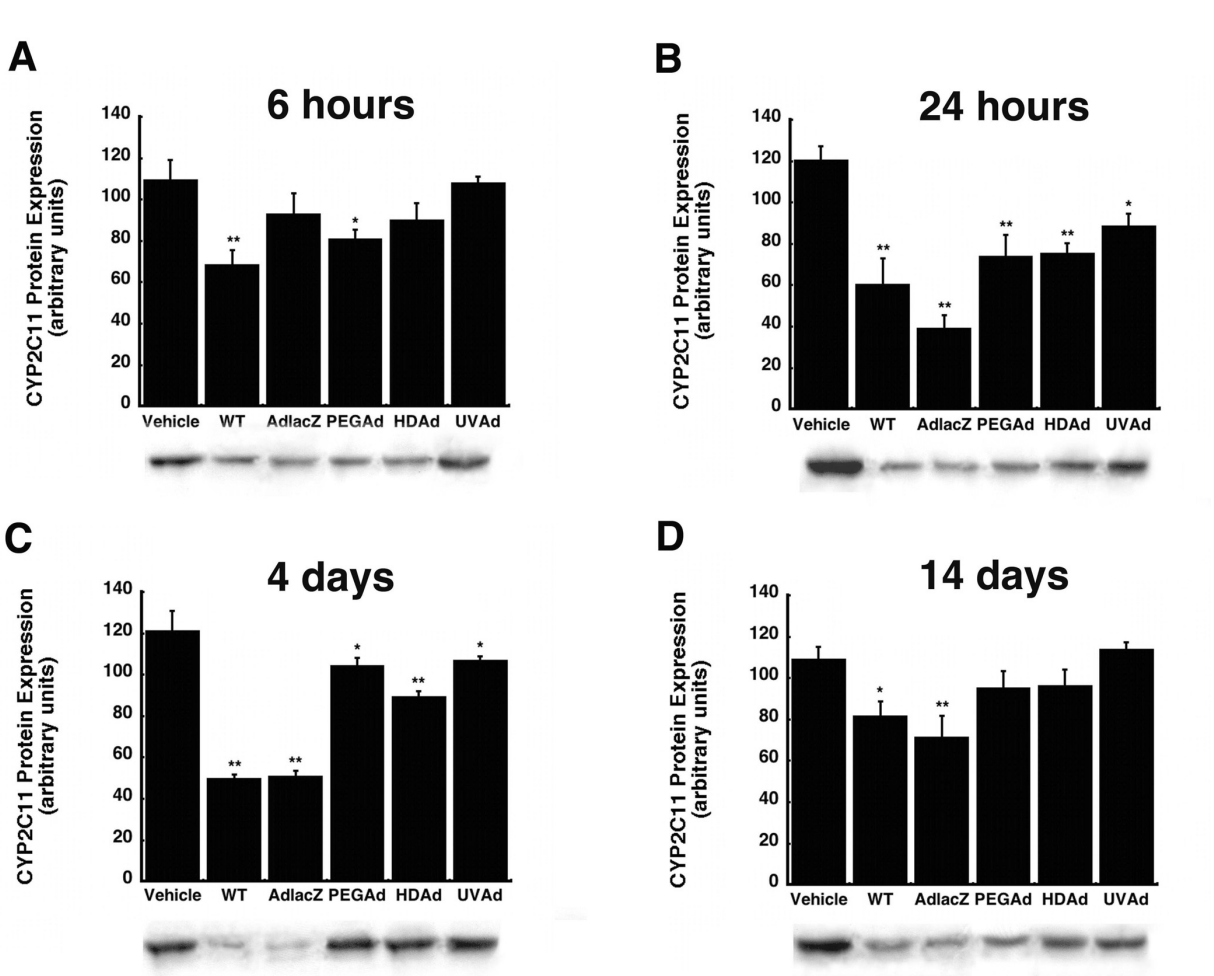
**A Single Dose of Adenovirus Significantly Suppresses Hepatic CYP2C11 1 and 4 Days After Administration**. Mean hepatic CYP2C11 protein expression levels 0.25 (A), 1 (B), 4 (C), and 14 (D) days after a single dose of: phosphate buffered saline (Vehicle), wild type adenovirus 5 (WT), first generation recombinant adenovirus expressing beta-galactosidase (AdlacZ), PEGylated AdlacZ, (PEGAd), helper-dependent adenovirus (HDAd), or inactivated virus (UVAd). Protein levels are reported as the relative density of positive bands with respect to that of a known protein standard in arbitrary units. Values are presented as the mean ± standard error of 4 animals/treatment/timepoint. Statistical significance was determined between individual treatment groups and vehicle controls by one-way analysis of variance with a Bonferroni/Dunn post-hoc analysis, *P ≤ 0.05 and **P ≤ 0.01.

CYP2C11 protein expression was significantly suppressed six hours after administration of the WT (37%) and PEGAd (26%) viruses (Figure 4A, *p *≤ 0.05). Twenty-two hours later, the most profound suppression (68% of control) was seen in animals given AdlacZ (Figure 4B, *p *≤ 0.01). At this time, this isoform was also significantly reduced by, 50, 38, 37, and 26% after treatment with WT, PEGAd, HDAd, and UVAd, respectively. CYP2C11 protein levels began to recover in animals treated with PEGAd, HDAd, and UVAd four days after administration, when they were only 14, 26, and 12% of saline treated controls (Figure 4C, *p *≤ 0.05). Protein levels continued to be markedly suppressed in animals given the WT (25%) and AdlacZ (35%) viruses fourteen days after treatment (Figure 4D, *p *≤ 0.05).

**Figure 5:**
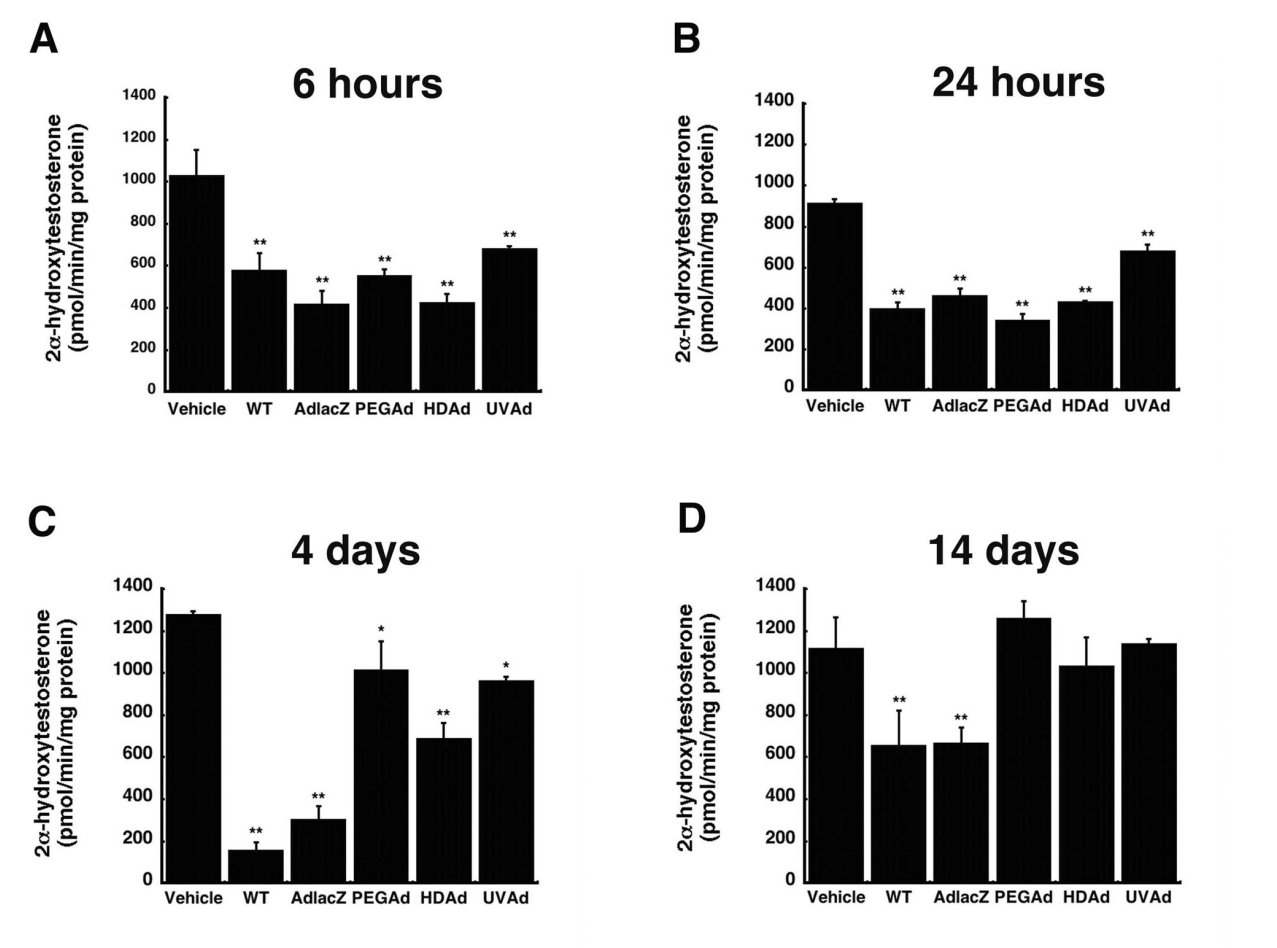
**A Single Dose of Active and Inactive Adenovirus Significantly Affects Hepatic CYP2C11 Catalytic Activity for Four Days**. *In vitro *catalytic activity of CYP2C11 microsomal proteins, as determined by measuring the production of the isoform-specific testosterone metabolite, 2α-hydroxytestosterone. Male Sprague-Dawley rats were given either: phosphate buffered saline (Vehicle), wild type virus (WT), first generation recombinant virus expressing beta-galactosidase (AdlacZ), PEGylated AdlacZ, (PEGAd), helper-dependent virus (HDAd), or inactivated virus (UVAd). Values are presented as the mean ± standard error of 4 animals/treatment/time point. Statistical significance was determined between each treatment group and vehicle controls by one-way analysis of variance with a Bonferroni/Dunn post-hoc test. *p ≤ 0.05, **p ≤ 0.01.

Each virus included in this study also significantly affected CYP2C11 activity, as determined by measuring the amount of the isoform-specific metabolite of testosterone, 2α-hydroxytestosterone, for fourteen days (Figure 5A, B, and 5C). Six hours after treatment, CYP2C11 activity was reduced by 34% in animals treated with UVAd and by approximately 52% in all other treatment groups (Figure 5A, *p *≤ 0.01). This effect persisted at the twenty-four hour time point when activity was 57, 50, 63, 53, and 26% of control (WT, AdlacZ, PEGAd, HDAd, and UVAd treatment groups, respectively, Figure 5B, *p *≤ 0.01). Four days after treatment, hepatic CYP2C11 activity was reduced by 88% and 76% in animals given WT and AdlacZ (Figure 5C, *p *≤ 0.01). This effect began to improve in animals given PEGAd (21%), HDAd (46%) and UVAd (25%) at the same timepoint (Figure 5C, *p *≤ 0.05) with complete restoration of normal CYP2C11 activity observed by day 14 (Figure 5D). At this timepoint, activity levels were still approximately 40% of control in animals given the WT and AdlacZ viruses (Figure 5D, *p *≤ 0.01).

**Figure 6:**
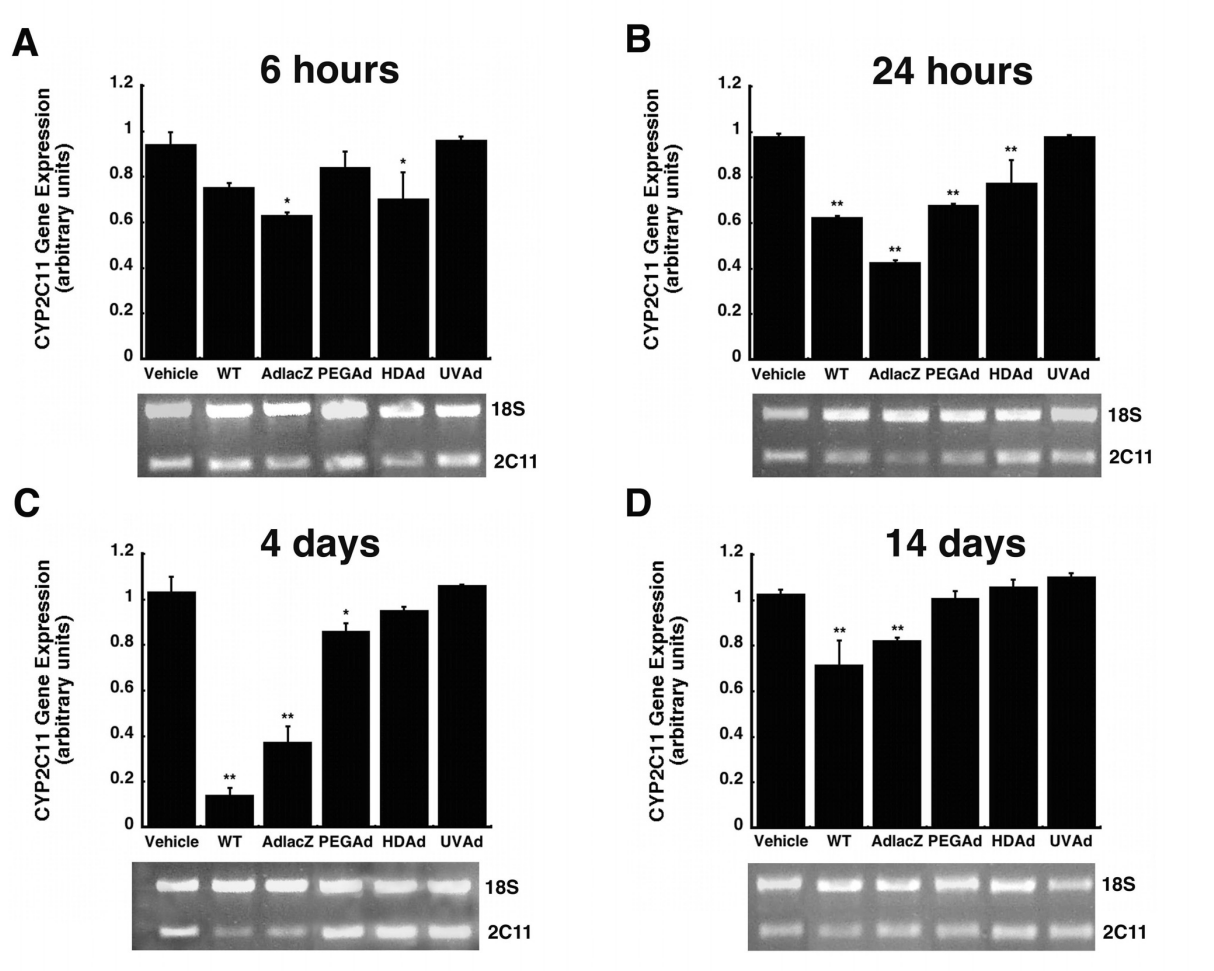
**Hepatic CYP2C11 mRNA levels are Significantly Reduced for at Least Four Days After A Single Dose of Active Adenovirus**. Mean relative intensities of RT-PCR products and representative agarose gels 0.25 (A), 1 (B), 4 (C), and 14 (D) days after treatment with wild type virus (WT), first generation recombinant adenovirus expressing beta-galactosidase (AdlacZ), PEGylated AdlacZ, (PEGAd), helper-dependent virus (HDAd), or inactivated AdlacZ (UVAd). mRNA levels are reported as band densities of gene-specific RT-PCR products with respect to the density of products from an internal control (18S rRNA) in arbitrary units. For all panels, animals dosed with phosphate buffered saline served as controls (Vehicle). Values are presented as the mean ± standard error of 4 animals/treatment/timepoint. Statistical significance was determined between individual treatment groups and vehicle controls by one-way analysis of variance with a Bonferroni/Dunn post-hoc analysis. *p ≤ 0.05, **p ≤ 0.01.

Treatment with AdlacZ and HDAd significantly reduced CYP2C11 mRNA levels six hours after administration (33 and 25% of control respectively, Figure 6A, *p *≤ 0.05). Changes in mRNA levels at the 24-hour time point closely mimicked that of protein expression in all treatment groups. The most significant reduction in hepatic CYP2C11 mRNA resulted from treatment with AdlacZ (56%) while the WT, PEGAd and HDAd treatments reduced mRNA by 36, 31, and 21% (Figure 6B, *p *≤ 0.01). By day four, animals treated with the WT virus experienced the most pronounced suppression (86%, Figure 6C, *p *≤ 0.01). At the same time, CYP2C11 mRNA was reduced by 63% and 15% in animals given the AdlacZ and PEGAd viruses (*p *≤ 0.05). By 14 days, mRNA levels in animals treated with PEGAd and HDAd had recovered to baseline while those treated with WT and AdlacZ continued to be suppressed by 29% and 18% respectively (Figure 6D, *p *≤ 0.01). No significant changes in CYP2C11 mRNA were detected in animals given the inactive virus (UVAd) throughout the course of the study.

### Assessment of serum ALT after a single dose of adenovirus

**Figure 7:**
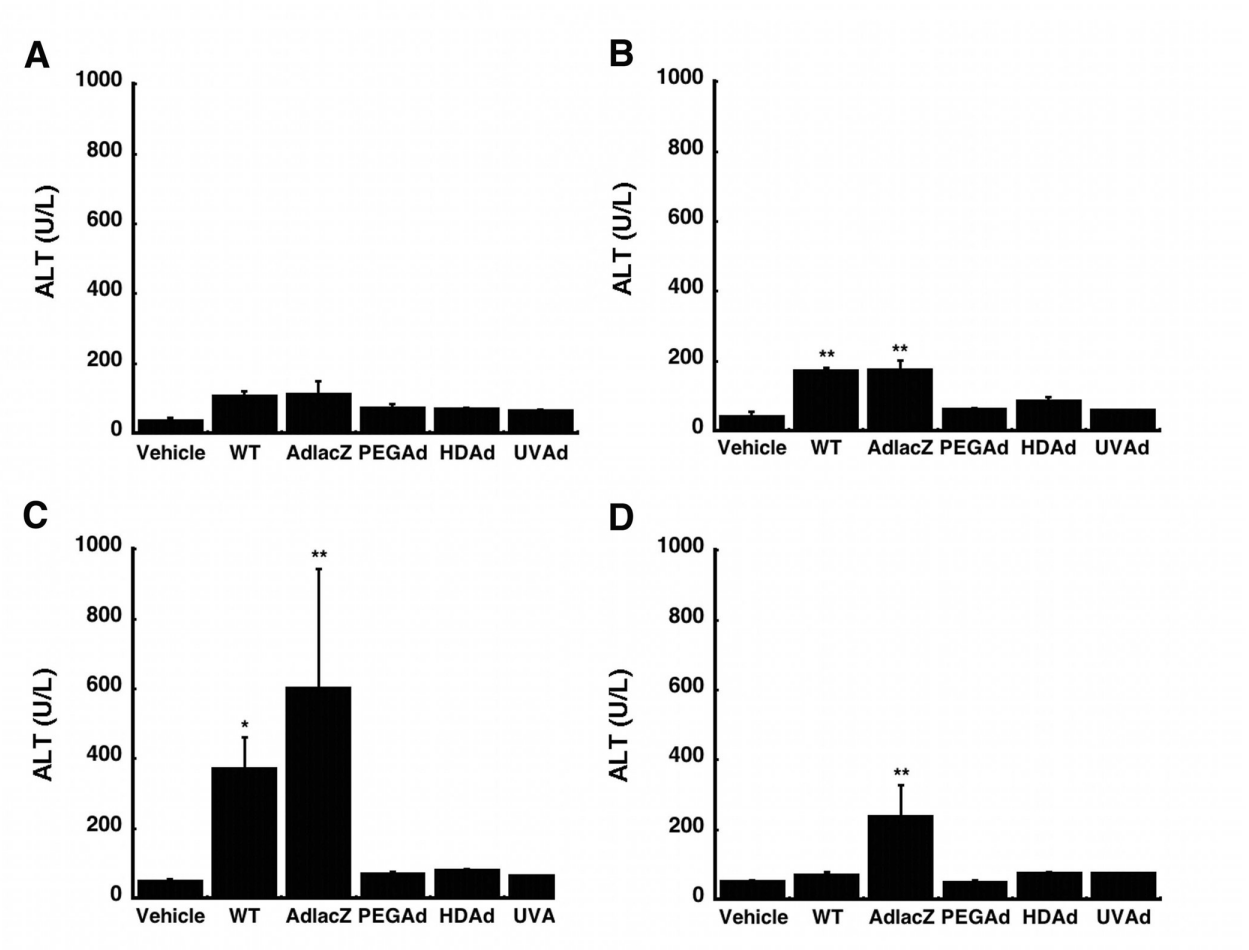
**Serum Transaminase Levels After A Single Dose of Adenovirus**. Alanine aminotransferase (ALT) levels following administration of: wild type adenovirus 5 (WT), first generation adenovirus expressing beta-galactosidase (AdlacZ), PEGylated AdlacZ, (PEGAd), helper-dependent virus expressing beta-galactosidase (HDAd), or inactivated virus (UVAd). For all panels, animals dosed with phosphate buffered saline served as controls (Vehicle). Values are presented as the mean ± standard error of 4 animals/treatment/timepoint. Statistical significance was determined between individual treatment groups and vehicle controls by one-way analysis of variance with a Bonferroni/Dunn post-hoc test. *p ≤ 0.05, **p ≤ 0.01

A dose-dependent, self-limiting hepatotoxicity, often indicated by transient elevation of serum transaminases, is known to occur soon after administration of recombinant adenoviruses [20,21]. In an effort to monitor the hepatotoxicity associated with each of the vectors included in this study, serum alanine aminotransferase (ALT) levels were assessed over 14 days. Only animals given WT or AdlacZ experienced significant changes in serum ALT (Figure 7). Twenty-four hours after administration, a 4-fold increase was observed in both treatment groups (Figure 7B, *p *≤ 0.01). Levels increased further by a factor of 7 (WT) and 12 (AdlacZ) above that observed in saline treated animals at the four-day time point (Figure 7C, *p *≤ 0.05). Fourteen days after administration, ALT levels returned to baseline in animals given the WT virus. Levels in animals treated with AdlacZ, however, remained elevated by a factor of 4.5 (Figure 7D, *p *≤ 0.05).

### Evaluation of transgene expression

**Figure 8:**
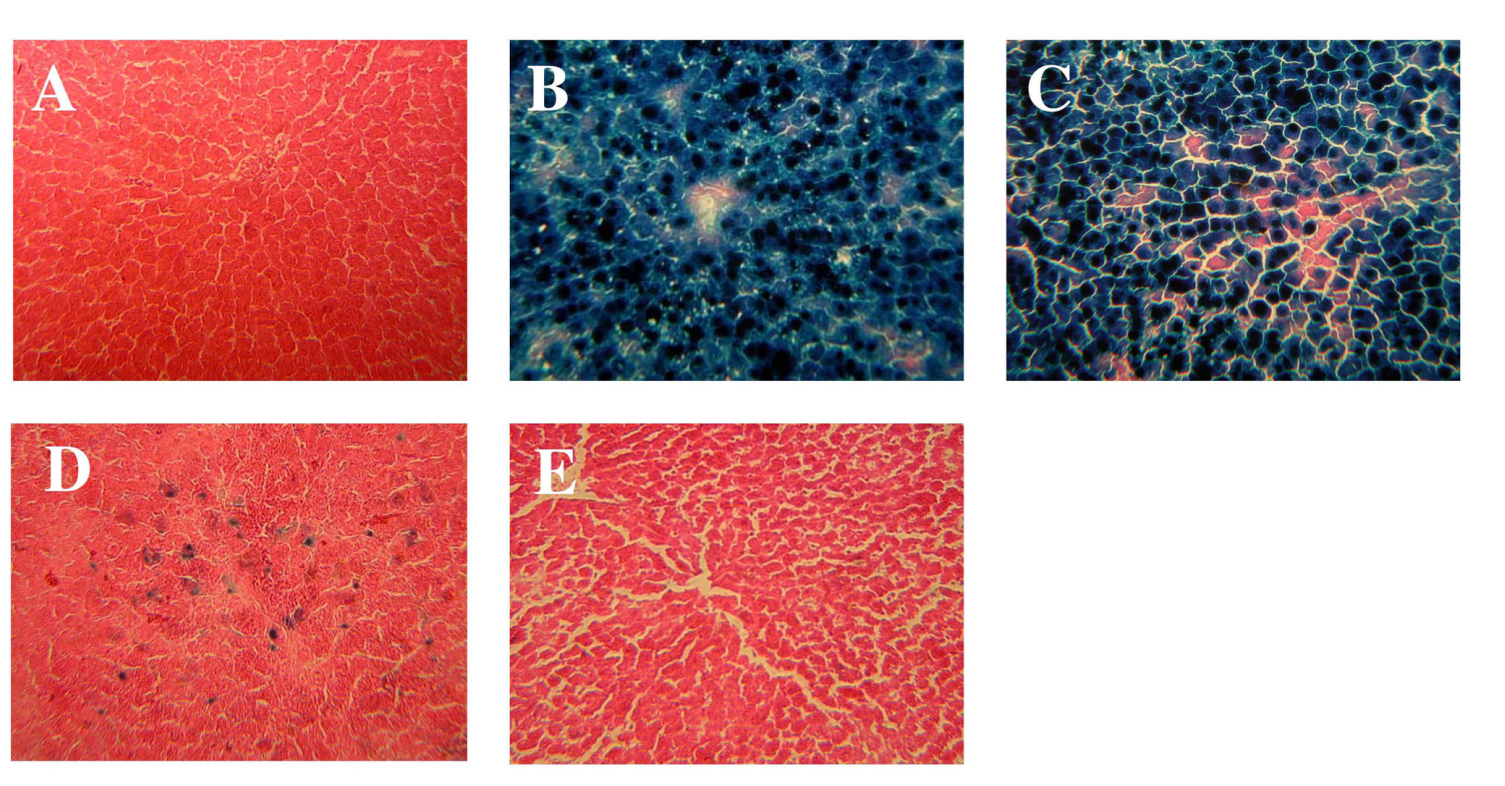
**Hepatic Localization of Transgene Expression Four Days After a Single Dose of Adenovirus**. Representative tissue sections isolated from animals given (A) phosphate buffered saline (B) first generation recombinant adenovirus 5 expressing beta-galactosidase (AdlacZ) (C) PEGylated AdlacZ (D) helper-dependent virus expressing beta-galactosidase or (E) inactivated virus. Original magnification of each panel: 200 ×.

Hepatic tissue isolated four days after treatment was sectioned and stained histochemically to evaluate the degree of transgene expression achieved with a single dose of each virus (Figure 8). Approximately 95% of hepatocytes expressed the beta-galactosidase transgene following administration of AdlacZ and PEGAd (Figure 8B and 8C). The HDAd vector transduced approximately 30% of all hepatocytes (Figure 8D). Complete inactivation of the AdlacZ vector was confirmed by the absence of beta-galactosidase expression in tissues isolated from animals given UVAd. These sections stained in a manner similar to that of saline treated animals (Figure 8E and 8A, respectively).

### Mechanistic evaluation of changes in CYP during viral infection

**Figure 9:**
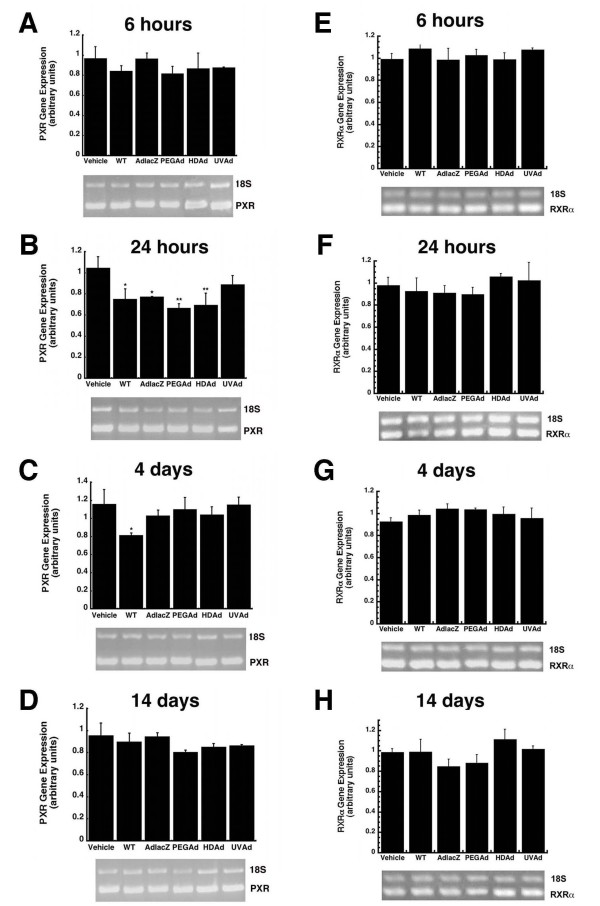
**A Single Dose of Recombinant Adenovirus Significantly Suppresses Pregnane × Receptor (PXR) mRNA in Male Sprague Dawley Rats 24 Hours After Treatment**. Mean relative intensities of RT-PCR products of PXR (Panels A-D) and the retinoid × receptor (RXR, Panels E-H) after treatment with either WT, AdlacZ, PEGAd, HDAd, or UVAd. mRNA levels are reported as band densities of gene-specific RT-PCR products with respect to the density of products from an internal control (18S rRNA). For all panels, animals dosed with phosphate buffered saline served as controls (Vehicle). Values are presented as the mean ± standard error of 4 animals/treatment/timepoint. Statistical significance was determined between individual treatment groups and vehicle controls by one-way analysis of variance with a Bonferroni/Dunn post-hoc test. *p ≤ 0.05, **p ≤ 0.01.

The pregnane × receptor (PXR) is a key regulator of CYP3A transcription and also contributes to CYP2C11 expression patterns [18,19,22]. Once activated, PXR forms heterodimers with the retinoid × receptor alpha (RXRα). This complex drives CYP expression. Hepatic PXR and RXRα mRNA levels were analyzed by RT-PCR in order to determine if our observations were due to changes in the expression of each of these key molecules during adenoviral infection. Twenty-four hours after administration, PXR levels were significantly suppressed in animals given the WT (28%), AdlacZ (26%), PEGAd (36%), and HDAd (33%) viruses with respect to vehicle treated controls (Figure 9B, P ≤ 0.05). Four days after treatment, PXR had returned to baseline in all treatment groups except those given the WT virus (30% of control, Figure 9C). PXR expression recovered to baseline by 14 days in all treatment groups (Figure 9D). Significant changes in RXRα expression were not detected in any of the treatment groups throughout the entire study (Figure 9E–9H).

## Discussion

Upsurges in Westerinization, urbanization and world travel have sparked similar trends in the exposure rate of the general public to infectious agents. Microbial infection can significantly compromise the expression and function of hepatic cytochrome P450 enzymes, responsible for catalyzing biochemical processes necessary to maintain physiological homeostasis and conversion of medicinal agents to pharmacologically or toxicologically active metabolites [5,6]. *In vitro *and *in vivo *studies suggest that cytokines and other immunoresponsive molecules associated with the acute phase of the immune response are largely responsible for this effect. We have found that a single dose of recombinant adenovirus significantly suppresses CYP3A2 and 2C11 expression and function in the male Sprague-Dawley rat for 14 days, long after the innate immune response against the virus resolves [9]. In an effort to prevent corresponding increases in drug-drug interactions, potential therapeutic failures of vital medications and secondary health problems due to interruption of natural biochemical processes during microbial infection, the experiments described in this manuscript were designed to determine how recombinant adenovirus alters CYP expression and function.

None of the modifications commonly made to reduce the immunogenicity and toxicity associated with adenoviral vectors completely resolved aberrations in hepatic CYP. Administration of helper-dependent adenovirus (HDAd), devoid of all viral genes and significantly less immunogenic than wild type or first generation adenoviruses [17,23], suppressed CYP3A2 protein, activity, and gene expression for 14 days (Figures 1, 2, 3, *p *≤ 0.01). Samples obtained from animals given the PEGylated virus, which is also significantly less immunogenic than the other viruses tested [15,16,24,25], followed a similar trend, suggesting that the immune response may not be the cause of suppression of this key hepatic isoform. At later timepoints, CYP mRNA levels were suppressed in animals given HDAd in a manner similar not only to the first generation virus, but to that of wild type virus, containing all viral genetic elements, suggesting that transcription and expression of viral genes cannot fully account for the observed reduction of CYP3A2. Administration of the helper-dependent and PEGylated viruses did, however, minimize changes in the expression and function of CYP2C11 with expression, activity, with mRNA beginning to recover four days after administration and resolving to baseline by day 14 (Figures 4, 5, 6, *p *≤ 0.05). This and data reported previously in which an E1/E3 deleted first generation adenovirus containing some viral gene expression elements but not a transgene cassette and another containing murine erythropoietin [10], further support the hypothesis that changes in the expression and function of CYP2C11 correlates with the immunogenicity of the vector and the trasngene constructs while changes in CYP3A2 may be due to shifts in cellular processes to support transgene production.

Many of the vectors employed in these studies had a mild toxicity profile with respect to serum alanine amintotransferase (ALT) levels. Only samples obtained from animals treated with the AdlacZ and WT vectors contained significant amounts of ALT (Figure 7). The PEGylated virus transduced hepatocytes in a similar manner to AdlacZ without affecting ALT, but still altered CYP expression and function (Figures 7 and 8). Treatment with the HDAd vector also did not affect ALT, but changes in CYP were still observed. Taken together, these results suggest that CYP alterations are not merely the result of hepatotoxicity arising from transgene product accumulation or overwhelming viral gene expression.

In an effort to identify a potential mechanism by which Ad infection alters CYP expression and function, we first chose to examine changes in expression of the pregnane × receptor (PXR). Transcription of CYP3A2 is thought to occur by heterodimerization of two orphan nuclear receptors, PXR and the retinoid × receptor alpha (RXRα) [19]. RXRα is a nuclear receptor that forms complexes with many other molecules to regulate gene transcription, whereas PXR has been often referred to as the "master" regulator of CYP3A [18,26-28]. PXR levels were significantly suppressed 24 hours after administration of all active recombinant viruses but recovered to baseline levels in all groups except those given wild type virus (Figure 9, *p *≤ 0.05). Given that CYP3A2 continued to be suppressed in all groups given active virus beyond 24 hours, we believe that changes in CYP3A2 expression and function may not be mediated by changes in PXR during adenovirus infection. This is further supported by another study in which it was reported that CYP was significantly suppressed in PXR knockout mice after treatment with bacterial endotoxin [29].

**Figure 10:**
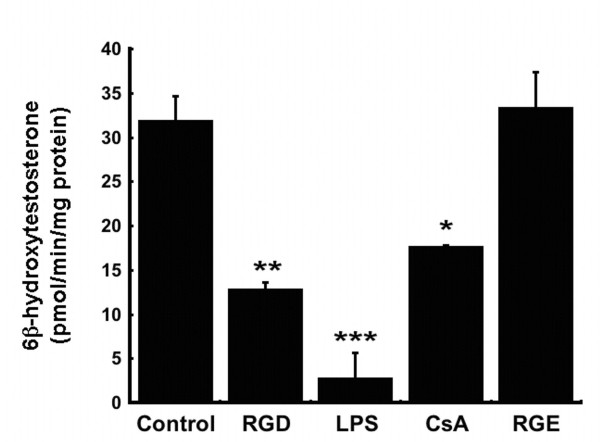
**Engagement of Integrin Receptors with a Non-Infectious Peptide Suppresses CYP3A2 in Primary Rat Hepatocytes**. Treatment with a peptide rich in RGD sequences at a concentration known to reduce adenovirus transduction by over 80% in culture (1.5 mg/ml, [58]) reduced CYP3A2 activity in primary hepatocytes by 60%. A peptide that differed by only one amino acid residue at the same concentration (RGE, 1.5 mg/ml) that does not block/engage integrin receptors did not affect CYP activity. Cells were also treated with bacterial lipopolysaccharide (LPS, 10 μg/ml) and cyclosporine A (CsA, 5 μg/ml), compounds that also suppress CYP3A2 [59,60], to confirm our results. Values are presented as the mean ± standard error of 3 replicates for each treatment group. Statistical significance was determined between individual treatment groups and vehicle controls by one-way analysis of variance with a Bonferroni/Dunn post-hoc test. *p ≤ 0.05, **p ≤ 0.01, ***p ≤ 0.001.

Although no appreciable changes in PXR and RXR mRNA levels were detected after administration of any of the vectors studied, post-translational modifications of these proteins and CYP itself such as phosphorylation, ubiquitination and redistribution between the nucleus and cytoplasm in response to virus-induced cell signaling cascades could account for the observed reduction in CYP during adenovirus infection and would not be readily detectable by the techniques used to assess changes in CYP, RXR and PXR described in this manuscript [30-32]. The possibility that these modifications are occurring and, in turn, impacting CYP expression and function is highlighted by the fact that administration of the UVAd vector significantly suppressed enzyme activity as early as six hours, an effect that lasted for 14 days in the case of CYP3A2 (Figure 2). This profound post-translational effect that a genetically inactive yet intact virus had on each CYP isoform may be the direct result of interaction of capsid proteins with virus receptors and subsequent internalization of virus particles at the cellular level. Binding of the fiber protein to the primary adenovirus receptor, the Coxsackievirus and Adenovirus receptor (CAR), facilitates interactions between penton base proteins and cell surface integrins promoting virus internalization [33]. Engagement of integrin receptors during this process initiates several signal transduction pathways such as extracellular-signal-regulated kinase (ERK), phophatidylinositol 3-kinase (PI3K) and protein kinases A and C (PKA and PKC) which may contribute to the observed changes in CYP after administration of any of the vectors included in this study [34-36]. Data from *in vitro *studies and microarray analysis of hepatic tissue provide further support that notion that engagement of integrin receptors, common points of entry for many pathogenic organisms [37], is sufficient to initiate changes in CYP3A2. Treatment of primary hepatocytes with a peptide known to block integrin receptors and prevent adenoviral infection significantly reduced CYP3A2 activity with respect to untreated controls (Figure 10).

Microarray analysis of hepatic tissue samples (oligo GEArray Rat Signal Transduction Pathway Finder microarrays; SuperArray, Frederick, MD) revealed that administration of each of the viruses significantly altered gene expression patterns associated with several key signal transduction pathways (data not shown). Expression of genes associated with the PI3K, PKC and nuclear factor kappa B (NFKB) pathways were induced with respect to those found in saline treated animals on average by a factor of 3.8, 4 and 4.6 respectively by each of the viruses included in this study. These pathways are of particular interest in the context of explaining our findings with respect to PXR and RXR expression. Ding and Staudinger described an increase in PXR activity in the presence of compounds that induced protein kinase A *in vitro *[38]. They subsequently found via a reporter gene assay that PXR activity was significantly suppressed after treatment with compounds that induced protein kinase C [39]. If this is the case *in vivo*, we believe that adenovirus infection did not significantly affect PXR levels because both PKA and PKC are upregulated during adenovirus infection [33,34], keeping the expression of this protein in check except at the 24 hour time point when the balance between the expression of each enzyme might be disrupted since they are each uniquely involved at different stages of virus internalization and trafficking to the nucleus which occur during this timeframe [34]. It has also been shown that activation of c-Jun N-terminal kinase (JNK), a kinase downstream of PKC [40], induces phosphorylation of RXRα [41,42], which causes it to redistribute from the nucleus to the cytoplasm, preventing it from forming the heterodimer complex with PXR and suppressing CYP expression and function [43]. Microarray data also suggests that changes in CYP after adenovirus infection may be in response to products associated with the NFKB pathway. The small heterodimer partner (SHP), an orphan nuclear receptor specifically expressed in the liver and a limited number of other tissues, is a transcriptional co-activator of NFKB and is also activated by JNK [44,45]. SHP can bind to both PXR and RXRα preventing heterodimer formation necessary for CYP expression [43,46-48]. To date an increase in SHP expression during adenovirus expression has not been described. Additional studies assessing the level of SHP in the liver during adenovirus infection, distribution patterns and phosphorylation status of PXR and RXRα *in vitro *and *in vivo *will further support these hypotheses and are currently underway in our laboratory.

## Materials and methods

### Materials

The following were purchased from Sigma-Aldrich (St. Louis, MO): phosphate-buffered saline (PBS), L-lysine, riboflavin, polyoxyethylene-sorbitan monolaurate (Tween 20), dimethylsulfoxide (DMSO), ethylenediaminetetraacetic acid (EDTA), formaldehyde, isopropanol, glucose-6-phosphate, β-nicotinamide adenine dinucleotide phosphate sodium salt (NADP^+^) and 11α-hydroxyprogesterone. Protogel^® ^acrylamide was purchased from National Diagnostics (Atlanta, GA). 5-bromo-4-chloro-3-indolyl-β-D-galactoside (X-gal) was purchased from Gold Biotechnology (St. Louis, MO). Polyclonal rat CYP3A2 and CYP2C11 primary antibodies were from BD Gentest (Woburn, MA). Corresponding horseradish peroxidase-conjugated secondary antibodies were from ICN Pharmaceuticals, Inc. (Aurora, OH). Isoform-specific CYP protein standards were from XenoTech, LLC (Lenexa, KS). PCR primers were purchased from Sigma-Genosys (The Woodlands, TX). Unless noted otherwise, all other materials were purchased in the highest purity from EMD Chemicals (Gibbstown, NJ).

### Adenovirus Production

WT (VR-5, ATCC, Manassas, VA) and first generation recombinant adenovirus 5 expressing the *E. coli *beta-galactosidase transgene under the control of a CMV promoter (AdlacZ) were amplified in 293 cells. Helper-dependent adenovirus 5 (HDAd) was prepared using the HD-Ad-SRα-β GEO vector containing a fusion gene of the *E. coli *beta-galactosidase transgene and the neomycin resistance gene under the control of the SRα promoter in 293Cre cells as described [49]. Amplification and rescue of the vector was achieved with the use of the AdLC8cLuc helper virus. Both vectors were purified from cell lysates by banding twice on cesium chloride gradients. AdlacZ was desalted on an Econo-Pac 10DG disposable chromatography column (BioRad, Hercules, CA) equilibrated with phosphate buffered saline, pH 7.4. HDAd was desalted by dialysis overnight in the same buffer. Contamination of helper virus was determined in the laboratory of Dr. Lucio Pastore, Federico II University, according to established techniques [50]. Positive fractions were collected and the number of virus particles (active and inactive) determined using the method of Maizel et al. with the following formula [51]:

Virus particles/ml = (absorbance at 260 nm) × (dilution factor) × 1.1 × 10^12^

All animals were treated with freshly purified virus.

### PEGylation of Adenovirus

Adenovirus expressing beta-galactosidase was prepared as described above. Protein content of the preparation was determined using BioRad DC Protein Assay reagents and bovine serum albumin as a standard in a microplate format. Ten micrograms of monomethoxypoly(ethylene) glycol, activated by tresyl chloride (Sigma Aldrich), was added for each microgram of protein present [52]. The coupling reaction was performed at 25°C with gentle agitation. The reaction was stopped by the addition of L-lysine, in a 10-fold excess with respect to the amount of PEG added. Unreacted PEG, excess L-lysine, and reaction byproducts were removed by buffer exchange over an Econo-Pac 10DG disposable chromatography column equilibrated with 100 mM potassium phosphate-buffered saline (pH 7.4).

### Riboflavin-Mediated Inactivation of Recombinant Adenovirus

Recombinant adenovirus 5 expressing beta-galactosidase was inactivated by a method unique to our laboratory as described [53]. A sufficient amount of riboflavin stock solution (1665 μM in DMSO) was added to purified virus to yield a final concentration of 50 μM. The virus/riboflavin mixture was placed in a 100 mm polystyrene dish (Fisher Scientific, Pittsburgh, PA) surrounded by ice and "sandwiched" between two UV light sources (Ultra-Lum, Claremont, CA and UVP, Upland, CA) each emitting UV light (365 nm, 1000 μW/cm^2^) for 45 minutes. Virus inactivation was confirmed by serial dilution of samples and infection of HeLa cells as described [53].

### Administration of Recombinant Adenovirus

All procedures were approved by the Institutional Animal Care and Use Committee of The University of Texas at Austin and are in accordance with the guidelines established by the National Institutes of Health for the humane treatment of animals. Male Sprague-Dawley rats (9–10 weeks old, Harlan Sprague Dawley, Inc. (Indianapolis, IN) were housed in individual cages and allowed unrestrained access to standard rodent chow (Harlan Teklad, Indianapolis, IN) and tap water. A single intra-muscular injection of a 1:1:1 (v/v/v) ratio of ketamine (100 mg/ml, Wyeth, Fort Dodge, Animal Health, Overland Park, KS), xylazine (20 mg/ml, Sigma Aldrich), and acetopromazine (10 mg/ml, Sigma Aldrich) achieved deep plane anesthesia for placement of catheters into the right jugular vein. Twenty-four hours after surgery, rats were given a single intravenous dose of 5.7 × 10^12 ^viral particles per kilogram (vp/kg) in a 0.5 ml volume of either: WT, AdlacZ, PEGAd, UVAd, or vehicle, phosphate buffered saline. A separate group was given 1.3 × 10^12 ^vp/kg of the HDAd vector in the same volume of saline. This dose was based upon the typical yield for this virus in our laboratory. Upon sacrifice, serum was collected for assessment of alanine aminotransferase (ALT). A small section of liver was immediately excised and stored in RNAlater™ (Qiagen, Valencia, CA) at 4°C for microarray analysis. Additional tissue was placed in Tissue-tek^® ^embedding medium (Fisher Scientific, Pittsburgh, PA) for X-gal histochemistry. Remaining tissue was excised, rinsed in saline, snap frozen in liquid nitrogen, and stored at -80°C for microsome preparation, and PCR.

### Isolation of Primary Hepatocytes

Hepatocytes were isolated from adult male Sprague Dawley rats (200–300 g) by a modified two step *in situ *collagenase perfusion protocol [54]. Cell isolates were further purified on Percoll gradients and seeded at a density of 1.5 × 10^5 ^cells/cm^2 ^onto rat tail collagen treated culture dishes (BD Biosciences, Bedford, MA). Cells were maintained in HepatoZYME-SFM (Invitrogen, Carlsbad, CA), supplemented with 1% L-glutamine (Hyclone, Logan, UT), gentamycin (0.5 μg/ml, Cambrex Biosciences, Walkersville, MD) and penicillin (100 U/ml)/streptomycin (100 μg/ml) (Mediatech, Herndon, VA).

### Microsome Isolation

Hepatic microsomal proteins were isolated by differential centrifugation as described previously [9]. Microsomes were stored at -80°C prior to analysis.

### Gel Electrophoresis and Immunoblot Analysis

Microsomal proteins (20 μg) were separated by size by sodium dodecylsulfate polyacrylamide gel electrophoresis (SDS-PAGE) as described [9]. Detection of putative proteins was achieved with a 1:3000 dilution of the specific primary CYP antibody in 3% NFDM followed by a second incubation with a corresponding horseradish peroxidase conjugated secondary antibody (1:3000). Immune complexes for CYP3A1/2 and CYP2C11 were detected by chemiluminescence (Western Lightning detection kit, PerkinElmer, Boston, MA). Protein band densities were analyzed using Kodak 1D image analysis software (Eastman Kodak, Rochester, NY). CYP3A1 and CYP3A2 co-migrate during electrophoresis. The antibody used to detect CYP3A2 was polyclonal with cross reactivity to CYP3A1, therefore all protein levels for CYP3A2 are reported as CYP3A1/2.

### *In vitro *Testosterone Hydroxylation Assay

Metabolic activity for CYP3A2 and 2C11 was determined by *in vitro *analysis of testosterone hydroxylation as described [9]. Samples were incubated with testosterone (Sigma Aldrich) for 18 minutes at 37°C with gentle agitation after addition of glucose-6-phosphate dehydrogenase (1 unit/μl, Sigma) and then quenched with dichloromethane (5 ml). 11α-hydroxyprogesterone (1.2 μg, Sigma) was added as an internal standard. The organic phase was evaporated under a constant stream of air, dissolved in 200 μl of methanol and stored in a sealed tube at 4°C until analysis. Testosterone metabolites were separated and quantified by HPLC according to a previously described method [55]. Peak areas of corresponding hydroxylation metabolites were measured and compared to peak areas of the internal standard within the same run.

### RT-PCR

Hepatic RNA was isolated with TRIzol (Invitrogen, Carlsbad, CA) according to the manufacturer's instructions. Samples were stored at -80°C prior to analysis. Hepatic RNA (1 μg) was generated using a reverse transcription kit, RETROscript, (Ambion, Austin, TX) following the manufacturer's instructions. PCR was performed using ReadyMix PCR Reaction Mix (Sigma-Aldrich). Each reaction (final reaction volume 12.5 μl) contained 0.5 μl of reverse-transcription product, 0.1 mM of each primer and 1 μl of QuantumRNA™ 18S internal standard (Ambion, Austin, TX) at a primer:competimer ratio of 4:6 for CYP 3A2 and 2C11 and 3:7 for PXR and RXR reactions. Gene-specific reaction conditions and primer sequences are summarized in Table 1. Reaction products were visualized on a 1.5% agarose gel containing ethidium bromide and band intensity determined using Kodak 1D image analysis software.

**Table 1:** Oligonucleotide primer sequences and amplification conditions for RT-PCR analysis of hepatic CYP and related nuclear receptors^a^

Gene	Sequence (5'-3')	PCR product (bp)	PCR conditions	
			
			Cycle	Annealing temperature (°C)
CYP3A2	Sense: TTG ATC CGT TGT TCT TGT CA Antisense: GGC CAG GAA ATA CAA GAC AA	323	23	60
CYP2C11	Sense: CTG CTG CTG CTG AAA CAC GTC Antisense: GGA TGA CAG CGA TAC TAT CAC	249	22	60
PXR	Sense: GAG CTC TGG GCA GAA ACA TC Antisense: ACA CGG CAG ATT TGA AGA CC	217	29	62
RXRα	Sense: CTC TAC CCA GGT GAA CTC TT Antisense: TGC TGC TCA CAG GGT TCA TG	293	30	58.4

^a^Primer sequences for RXRα were obtained from Macejova et al. [57]. All other sequences were designed in our laboratory as described previously [9].

### Liver function analysis

Serum ALT levels were measured with Vitros ALT slides on a Vitros DT60 autoanalyzer (Ortho-Clinical Diagnostics, Rochester, NY).

### X-Gal histochemistry

Frozen liver sections (6 μm) were fixed in 0.5% glutaraldehyde and stained for beta-galactosidase activity as previously described [56].

### Data analysis

One-way analysis of variance with a Bonferroni/Dunn post-hoc analysis was used to determine statistical differences between individual groups (SuperANOVA, Abacus Concepts, Berkley, CA). Differences were determined to be significant when the probability of chance explaining the results was reduced to less than 5% (*p *< 0.05).

## Competing interests 

The authors declare that they have no competing interests.

## Authors' contributions

SMC performed all *in vivo *experiments, analyzed data and wrote the manuscript; PW assisted with virus production, performed RT-PCR on samples collected from *in vivo *studies, conducted all *in vitro *studies in primary hepatocytes, and analyzed data. MAC conceived the study design, assisted with virus production and animal experiments, supervised the project and wrote the manuscript.
